# STAT3 and p63 in the Regulation of Cancer Stemness

**DOI:** 10.3389/fgene.2022.909251

**Published:** 2022-08-17

**Authors:** Shixiong Wei, Jialin Li, Mingbo Tang, Kewei Zhang, Xinliang Gao, Linan Fang, Wei Liu

**Affiliations:** Department of Thoracic Surgery, The First Hospital of Jilin University, Changchun, China

**Keywords:** STAT3 (signal transducer and activator of transcription 3), P63 (TP63), cancer, stemness, cancer stem cell

## Abstract

Signal transducer and activator of transcription 3 (STAT3) is a transcription factor with many important functions in normal and transformed cells. STAT3 regulatory activities are highly complex as they are involved in various signaling pathways in different cell types under different conditions. Biologically, STAT3 is a regulative factor for normal and cancer stem cells (CSCs). Tumor protein p63 (p63), a member of the p53 protein family, is involved in these biological processes and is also physically and functionally associated with STAT3. STAT3 activation occurs during various aspects of carcinogenesis, including regulation of CSCs properties. In combination with p63, STAT3 is a possible biological marker of CSCs and a major regulator of maintenance of stemness in CSCs. We summarized the STAT3 functions and regulation and its role in CSC properties and highlight how these are affected by its associations with p63.

## 1 Introduction

Tumor protein p63 (p63), a member of the p53 protein family, has been associated with Signal transducer and activator of transcription 3 (STAT3), which is one of the seven members of the STATs family of transcription factors (Galoczova et al., 2018a; Wei, 2020). As essential cell survival and proliferation regulators, both STAT3 and p63 have crucial functions in maintenance of stem cell stemness and differentiation. Moreover, they are involved in carcinogenesis of numerous cell types. STAT3 regulates cancer suppressor genes and oncogenes and influences tumor microenvironments (Zou et al., 2020). P63 is commonly associated with epithelial malignancies, particularly squamous cancers (Gatti et al., 2022). Functionally, p63 is also essential for cell adhesion and motility and plays significant roles in regulating various genes that are involved in tumor proliferation, survival, and differentiation (Gatti et al., 2019). In this review, STAT3, its regulation, its roles in cancer stemness maintenance, and its relationship with p63 are elucidated.

## 2 Structure and Regulation of STAT3

**FIGURE 1:**
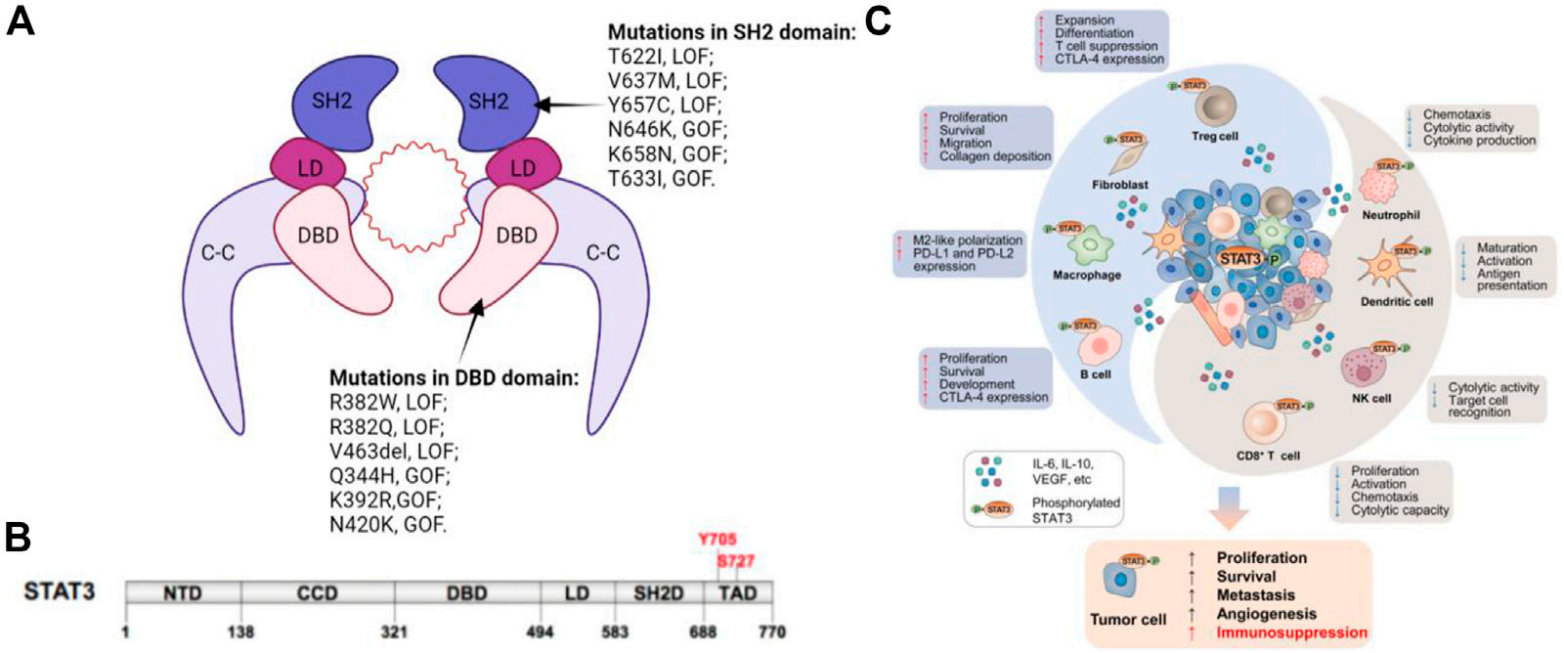
**(A)** Schematic illustration of STAT3 dimer bound to DNA. This is a representation of the major STAT3-DNA binding complex (resolved in the X-ray crystal structure). **(B)** Primary structure of STAT3 showing domain organization along the polypeptide chain. **(C)** STAT3 induces immunosuppression in the TME. STAT3 activities in tumor cells augment cancer hallmarks, including increased secretion of immunosuppressive factors, such as IL-6, IL-10, and EGFR, which can activate STAT3 in innate and adaptive immune cell subsets as well as CAFs in the TME. Immune cells and CAFs within the TME can release certain factors, including IL-6, which enhances STAT3 signaling in tumor cells. Elevated STAT3 in the TME has dual effects.

The STATs family members have similar functional domains, that is, N-terminal domain, central DNA-binding domain, and coiled-coil domain that can achieve protein-protein interactions as well as linker domain that can influence DNA-binding stability and classic SRC homology2 (SH2) domain. STAT3 has two significant phosphorylation positions, a tyrosine residue at amino acid position 705 (Tyr705) within the SH2 domain and a serine phosphorylation site at position 727 (Ser727) within the C-terminal transactivation domain, that are however absent in alternatively spliced STAT3-β variants (Figure 1) (Galoczova et al., 2018a).

Functional diversity of STAT3 in different cell types is demonstrated by its regulation of numerous activators and negative regulators. Phosphorylation of Tyr705 rather than Ser727 by upstream kinases is the main mechanism of STAT3 activation. Non-phosphorylation mechanisms through which STAT3 activation is regulated include post-translational modifications such as acetylation, methylation, and ubiquitination. Negative regulation of STAT3 activation occurs via protein phosphatases, specific protein inhibitors-suppressors of cytokine signaling (SOCS), protein inhibitors of activated STAT (PIAS), and several miRNAs (Butturini et al., 2020). Activation and expressions of STAT3 are regulated by various signals, which function in multiple signaling pathways, thereby making STAT3 a flexible and adaptable regulator that directly and indirectly regulates gene expressions in different types of cells under diverse conditions (Tošić and Frank, 2021).

## 3 STAT3, CSCs, and Cancer Stemness

**FIGURE 2:**
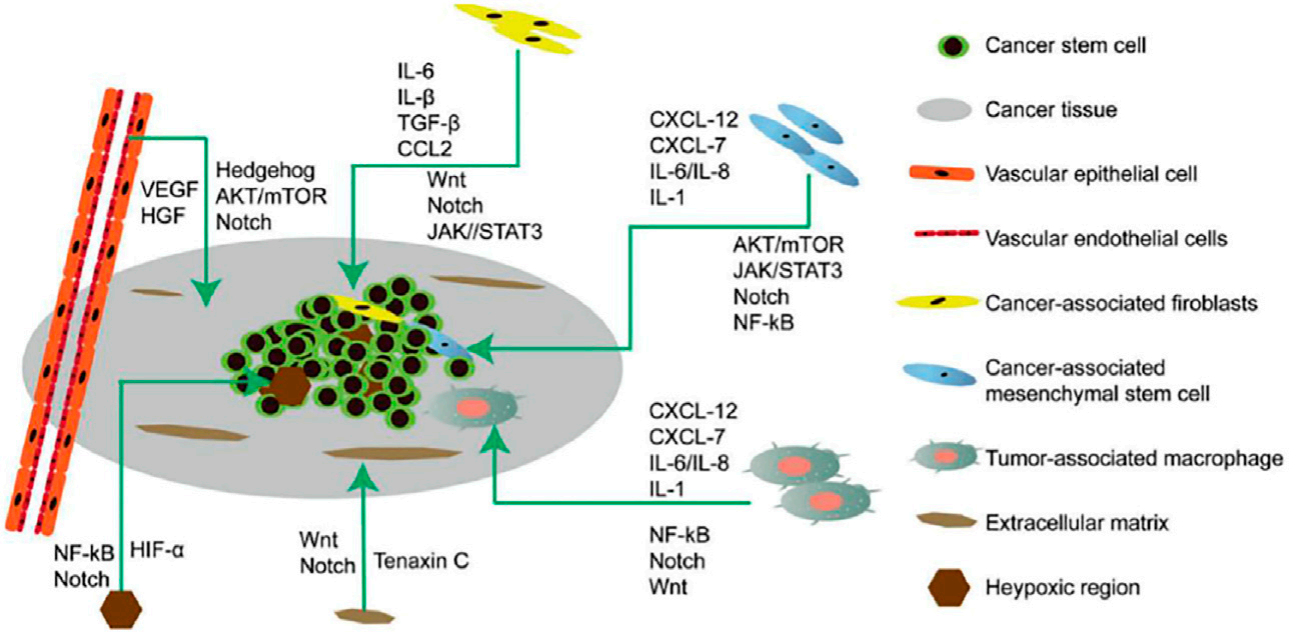
Microenvironment of cancer stem cells (CSCs) illustrating their proliferation, self-renewal, differentiation, metastasis, and tumorigenesis. The CSC microenvironment is mainly composed of vascular niches, hypoxia, tumor-associated macrophages, cancer-associated fibroblasts, cancer-associated mesenchymal stem cells, and an extracellular matrix. These cells, in response to hypoxic stress and matrices, induce growth factors and cytokines (such as IL-6 and VEGF) to regulate CSC growth *Via* Wnt, Notch, and other signaling pathways.

Cancer stem cells (CSCs) were first isolated from leukemia cells and identified as therapeutic targets for cancer. CSCs undergo self-renewal, are pluripotent, and are involved in recurrence, metastasis, heterogeneity, and drug and radiation resistance of tumors (Yang et al., 2020). CSCs are regulated by pluripotent transcription factors (OCT4, Sox2, Nanog, KLF4, and MYC). They are also regulated by intracellular signaling pathways, including Wnt, NF-κB, Notch, Hedgehog, JAK-STAT, PI3K/AKT/mTOR, TGF/SMAD, PPAR, and extracellular factors, such as vascular niches, hypoxia, tumor-associated macrophages, cancer-associated fibroblasts, cancer-associated mesenchymal stem cells, extracellular matrices, and exosomes. Thus, to potentially destroy CSCs, drugs, vaccines, antibodies and CAR-T cells that target these pathways are effective (Figure 2) (Liu et al., 2019).

Some of the STAT3-associated signaling pathways have been reported in some hematological malignancies and solid tumors (Butturini et al., 2020) and in facilitation of cancer proliferation via regulation of CSC activities. Activation of STAT3 is predictive of poor prognostic outcomes in many cancers. Even though STAT3 is rarely altered by changes in gene expressions, its mRNA levels usually present a similar trend with its activation (Heichler et al., 2020; Ma et al., 2020). STAT3 is involved in maintenance of expressions of genes that encode the stem cell phenotype and is, therefore, used as a CSC maker. Hence, the STAT3 pathway is active in cells that are rich in other CSC markers, and its inhibition—proteins that facilitate cell growth and proliferation reduced activities on the STAT3 pathway—potentially downregulates cell viability and tumorsphere formation (Gatti et al., 2019; Siersbæk et al., 2020).

The relationship between epithelial-mesenchymal transition (EMT) and tumor microenvironments is based on plasticity between CSCs and their more differentiated derivatives, in which differentiation occurs from CSCs to non-CSCs and vice versa with the reverse requiring EMT. STAT3 activation is involved in induction of EMT and such activation is via either IL-6 dependent or independent procedures, for instance, non-canonical Frizzled 2 pathway/TGF-β/LIF pathway (Jin, 2020). EMT induction and proliferation of CSCs, post STAT3 activation, were associated with resistance to trastuzumab or cisplatin (Chung et al., 2014). An IL-6 loop, in which IL-6 activates Akt, STAT3, and NF- κB pathways, and inhibits PTEN expressions has been documented (Galoczova et al., 2018a; Ma et al., 2020; Wei, 2020).

In the tumor microenvironment, STAT3 regulates NF-κB signaling in tumor and non-transformed stromal cells. Physically, STAT3 interacts with NF in tumor and tumor-associated immune cells (Sun et al., 2021), wherein lactoglobule-EGF Factor 8 (MGF-E8)/STAT3, Sonic Hedgehog/EGFR/STAT3/Sox2 pathways, and CSC-like phenotypes were promoted by tumor-associated macrophages (Wei, 2020; Gatti et al., 2022).

In conclusion, STAT3 has a complex role in maintaining and promoting CSC characteristics. Directly, STAT3 interacts with transmembrane glycoproteins expressed by normal stem cells and is a biomarker for CSC identification and isolation. EMT, as a mechanism by which CSCs are engendered, is correlated with STATs-associated pathways. STAT3 is critical in angiogenesis and regulation of the tumor microenvironment, which provides signals for differentiation or proliferation via inflammation, for instance, in the NF-κB pathway. Moreover, feedback activation of STAT3 is involved in anticancer drug resistance (Johnson et al., 2018; Zou et al., 2020).

## 4 Structure and Regulation of p63

P63, also known as tumor protein 63 (TP63) or transformation-related protein 63 (Trp63) or amplified in squamous cell carcinoma (AIS), is a member of the p53 transcription factor family and the corresponding p53/p63/p73 gene family that encodes p53, p63 and p73 proteins (Soares and Zhou, 2018; Fisher et al., 2020). These family members contribute to tumorigenesis as well as morphogenesis and have a similar domain organization—DNA binding domain (DBD), C-terminal oligomeric domain (OD), and N-terminal transcriptional activation domain (TAD). Structurally, they are tetramers and may form heterotetramers, because of their partial homology in oligomeric domains. P63 has numerous isoforms that are linked to stem cell development and differentiation, aging, proliferation, stem cell maintenance, senescence, and apoptosis (Galoczova et al., 2018b).In addition, by directly regulating chromatin-modulating factors, engaging and opening chromatin regions, p63 is essential for adjusting the chromatin landscape in epidermal keratinocytes (Galoczova et al., 2018b). P63 can bind target genes of p53 and p73 since all three have highly homologous DNA binding domains (Peng et al., 2021).

The TP63 gene encodes two types of isoforms and consists of 15 exons distributed across 270 kb of chromosome 3q27. Exon 1, which is found upstream of p63, is a transcription promoter of TAp63. TAp63 transcripts contain three TA-specific exons (Exons 1, 2, and 3) that encode N-terminal transactivation domains, homologous to those of p53 (Murray-Zmijewski et al., 2006; Tošić and Frank, 2021). β and δ isoforms lack exon 13 while γ lacks exons 11-14 but has a γ-specific exon 10′. Premature stop codon on exon 10 generates an ε isoform. The above isoforms have DNA-binding and oligomerization domains. TAp63 and ΔNp63 isoforms differ in organ sites of transcription, such that TAp63 transcripts are prevalent in the heart, kidney, brain, thymus, testis, and cerebellum, whereasΔNp63 transcripts are highly detected in the epithelia, spleen, kidney, and thymus (Mangiulli et al., 2009; Jin, 2020).

P63 regulates DNA damage responses, and this is both isoform and cell type specific. For instance, in response to DNA damage, TAp63 is expressed in epithelial tissues, neurons, and germlines. Expressions of ΔNp63 are induced by tyrosine kinase receptor epidermal growth factor receptors (EGFR). Unlike EGFR activation of phosphatidylinositol 3-kinase (PI3K) signaling in keratinocytes, EGFR activation of ΔNp63α is mediated by STAT3 in cancer, and these two pathways are potentially linked via the mammalian target of rapamycin (mTOR) as PI3K activation of mTOR results in mTOR-dependent activation of the STAT3/p63/Jagged pathway. Apart from EGFR-mediated activation of ΔNp63α, ΔNp63α expressions are induced by interactions of α6β4 integrin and specific proteins, such as transglutaminase 2 (TG2) (Fisher et al., 2016; Wei, 2020; Sun et al., 2021).

Wnt/β-catenin signaling is a highly conserved pathway that is involved in regulation of cellular proliferation, differentiation, migration, apoptosis, and stem cell renewal. Through the Wnt/β-catenin pathway, binding lymphoid enhancer-binding factor 1 (Lef1) to β-catenin between TAp63 and ΔNp63 promoters directly regulates p63 (Ferretti et al., 2011). As a downstream target of p63, Hedgehog signals are involved in embryonic development, organismal polarity formatting, wound healing, maintenance of somatic stems, and pluripotent cells. Reduced expressions of p63 are due to overexpression of the p65 subunit of NF-κB, possibly via NF-κB-mediated enhancement of expressions of the zinc finger E-box binding homeobox (ZEB1) (Wu et al., 2009).

## 5 Associations Between STAT3 and p63

The *TP63* gene is localized on chromosome 3 and gives rise to multiple isoforms due to differential promoter selection (full-length TA and N-terminal truncated ΔNp63) and alternative splicing of the 3′end of the mRNA (α, β, γ, δ, ε) (Yang et al., 1998). ΔNp63 isoforms lack the N-terminal transactivation domain, hence they are able to antagonize full-length isoforms of p63 as well as other p53 family members and act like dominant negative transcription inhibitors. However, they also have transactivation activities that are attributed to the presence of an alternative TAD (Yang et al., 1998; Chu et al., 2008). Among the C-terminal isoforms, p63α isoforms have a sterile alpha motif (SAM) that is involved in protein-protein interactions and have a transcription inhibitory domain (TID), which inhibits its transcriptional activities (Sayan et al., 2007; Rufini et al., 2011).

P63 is involved in epidermal development because it is highly expressed in basal cells of various epithelial tissues, thereby conferring them with stem-cell-like properties. ∆ p63 mice mutants either lack stratified squamous epithelia and their derivatives or have stratified but disrupted epidermis. Truncated or lost limbs and craniofacial abnormalities also characterize ∆ p63 mice mutants (Yang et al., 1998). Thus, basal cells are multipotent tissue-specific epithelial progenitors that express p63, cytokeratin 5, and cytokeratin 14. Like stem cells, these basal cells quickly proliferate in response to epithelial damage and contribute to the repair of damaged epithelium in both mouse and human trachea. P63 is a critical mediator of normal epithelial development, maintenance and homeostasis.ΔNp63α is a predominant p63 isoform in epithelial tissues and is highly expressed in basal cells of stratified and glandular epithelia, including epidermis. Its levels decrease with cellular differentiation. Conversely, TAp63 positive cells are lowly expressed in the stratified epithelia, indicating a switch between isoforms during differentiation (Galoczova et al., 2018a; Liu et al., 2019; Wei, 2020). As expressions of ΔNp63 proteins are only observed in other basal cells, such as breasts, prostate, bladder, and colorectum, they have been used as basal cell markers (Galoczova et al., 2018a; Wei, 2020). A possible association between p63 and activated STAT3 is the function of the latter in promoting airway ciliated cell regeneration from basal stem cells and malignant transformation of foregut basal progenitor cells. In addition, p63 works in tandem with STAT3 in human keratinocytes, as revealed via ChIP-Seq analysis (Sethi et al., 2014; Zou et al., 2020).

**FIGURE 3:**
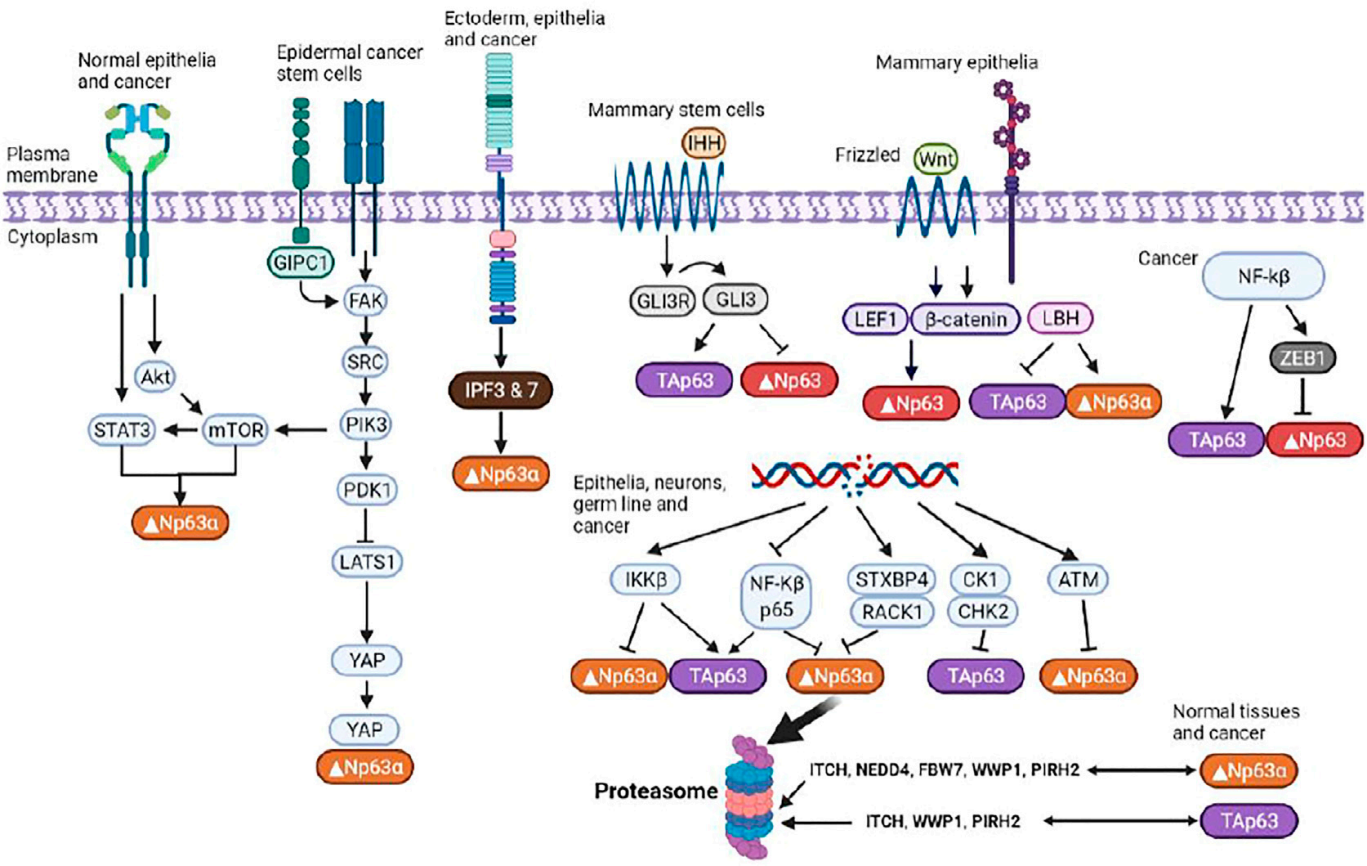
Regulation of p63 isoforms. P63 is involved in modulation of the chromatin landscape in epidermal keratinocytes by directly regulating chromatin-modulating factors and engaging and opening chromatin regions. Multiple isoforms with distinct, often opposing, functions enable p63 to exert an array of effects on essential cellular functions.

Since TAp63 and Δ Np63 oppose each other’s regulatory roles, P63 exhibits bidirectional regulation of tumorigenesis. Like STAT3, p63 is a highly conserved protein and its mutations, such as gene amplification, result in cancerous cells, in which p63 activities are increased (Koster et al., 2006). ΔNp63 is a putative oncoprotein whose expression is upregulated in squamous cell carcinomas, triple-negative basal-like breast tumors, and other tumor types. It also affects the various signaling pathways that contribute to development of CSC phenotypes (Gatti et al., 2022; Butturini et al., 2020). Moreover, apart from p53, TAp63 induces cell cycle arrest and apoptosis (Figure 3). ΔNp63 enhances the expressions of Wnt receptor Frizzled 7, thereby promoting Wnt signaling, leading to promotion of normal mammary stem cell activities and tumor-initiating activities in the basal-like subtype of breast cancer (Chakrabarti et al., 2014). Memmi et al. (Jin, 2020) revealed positive modulation of the Hedgehog signaling pathway by ΔNp63 that maintains the self-renewal potential of mammary CSCs. Autoregulation of ΔNp63 gene transcription is mediated by activation of STAT3 and its subsequent binding to STAT3RE. Since STAT3 activation by interleukin-6 also leads to Δ Np63 upregulation and blockade of either DeltaNp63 or STAT3 expressions by siRNA, it results in cell growth suppression. Thus, the identified regulatory pathway is of probable cell physiological significance.

**FIGURE 4:**
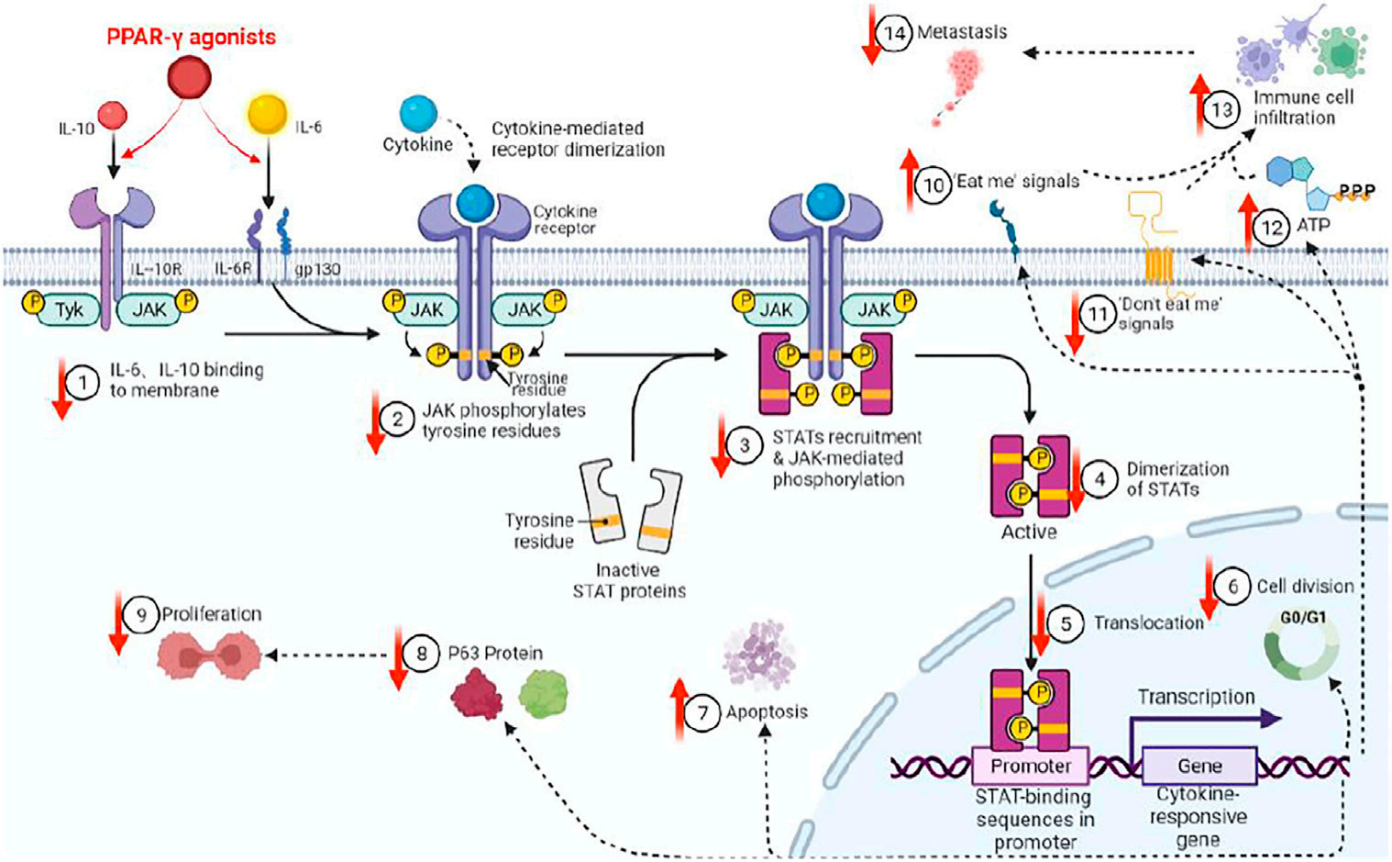
PPAR-γ agonist inhibits JAK-STAT signaling pathway activations through molecules such as IL6/IL10, thereby inhibiting p63 protein expressions in CSCs. We previously showed that PPAR-γ agonists target and inhibit CSC proliferation by downregulating the phosphorylation of the JAK-STATs pathway and p63 protein expressions, etc. (↓=downregulated, ↑=upregulated).

There are various associations between STAT3 andΔNp63. First, both are CSC markers that are associated with triple-negative breast tumors, which, when compared to non-triple negative types, had more CSC markers (Wei, 2020; Galoczova et al., 2018a; Gatti et al., 2022). Second, STAT3 is constitutively activated in squamous cell carcinomas, in which ΔNp63 is overexpressed. Third, both are the main regulators of CSC maintenance (Gerbe et al., 2019). Fourth, dual-regulatory effects of ΔNp63 on its own promoter are dependent on STAT3 activation, which binds the ΔNp63 promoter. Fifth, since the expressions of ΔNp63 are regulated by the EGFR/STAT3 axis that is vital for the proliferation of CSCs, activations, and expressions of both STAT3 andΔNp63 are possibly regulated in part by the EGFR signaling pathway (Ma et al., 2010). Sixth, we previously determined that peroxisome proliferator-activated receptor-γ(PPAR-γ) agonists inhibit stemness of lung adenocarcinoma cancer stem cells (CSCs) by downregulating the expressions of Janus tyrosine kinase (JAK)-signal transducer and activator of transcription (STAT) signaling pathway and elucidated p63-STATS connections are illustrated (Figure 4) (Wei, 2020).

Seventh, mTOR activates STAT3 to induce p63 expressions, which then activates Notch signaling by stimulating Jag1 gene expressions that inhibit murine and human cell differentiation (Lin et al., 2021). Peng et al. (2021) found that a sharp rise of IL-6 boosted STAT3 phosphorylation and then restrained p63 expression in bleomycin-induced acute lung injury. Further results suggested that mesenchymal stem cells' supernatant lyophilized powder activated p63 by inhibiting the IL-6-p-STAT3 pathway. Then, to clarify the role of IL-6, the researchers instilled rh IL-6 into the airways of mice. ELISA demonstrated that rh IL-6 was enriched in the lung tissues on day 1, after which it was absorbed and removed on day 2. Consistently, rh IL-6 administration promoted STAT3 phosphorylation and decreased p63 expression. Despite these close associations between STAT3 and ΔNp63, only ΔNp63 is involved in the inflammatory NF-κB pathway, angiogenesis through VEGF, and EMT—Δ Np63 increased EMT and reduced the opposing process of mesenchymal-epithelial transformation (MET). Hypothetically, p63 activates the Notch signaling pathway in adjacent cells, which increases EMT. Relatedly, TAp63 possibly contributes to cancer cell transitions to tumor-initiating cells.

Due to the abundance of possible interactions among p53, p63, and p73, studies on relationships between p63 and STAT3 and the role of p63 in cancer should also consider the expressions of the p63 isoforms and the relationship of these isoforms to other p53 family members.

## 6 Conclusion

STAT3 signaling is the main regulatory pathway for the fate of embryonic stem cells and the limiting factor of human somatic cell reprogramming. During carcinogenesis, STAT3 signaling activation is involved in many aspects, including regulating the properties of CSCs. STAT3 and p63 are the main regulators of CSC maintenance. Apart from being biomarkers of CSC, STAT3, and ΔNp63 interact directly and, through numerous ways, regulate CSC properties. More studies should be performed to elucidate on the associations between the two.

## References

[B1] Butturini E. Carcereri de Prati A. Mariotto S. (2020). Redox Regulation of STAT1 and STAT3 Signaling. Int. J. Mol. Sci. 21 (19), 7034. 10.3390/ijms21197034 PMC7582491 32987855

[B2] Chakrabarti R. Wei Y. Hwang J. Hang X. Andres Blanco M. Choudhury A. (2014). ΔNp63 Promotes Stem Cell Activity in Mammary Gland Development and Basal-like Breast Cancer by Enhancing Fzd7 Expression and Wnt Signalling. Nat. Cell. Biol. 16 (10), 10041–101513. 10.1038/ncb3040 PMC4183725 25241036

[B3] Chu W.-K. Dai P.-M. Li H.-L. Chen J.-K. (2008). Transcriptional Activity of the ΔNp63 Promoter Is Regulated by STAT3. J. Biol. Chem. 283 (12), 7328–7337. 10.1074/jbc.M800183200 18198175

[B4] Chung S. S. Giehl N. Wu Y. Vadgama J. V. (2014). STAT3 Activation in HER2-Overexpressing Breast Cancer Promotes Epithelial-Mesenchymal Transition and Cancer Stem Cell Traits. Int. J. Oncol. 44 (2), 403–411. 10.3892/ijo.2013.2195 24297508 PMC3898805

[B5] Ferretti E. Li B. Zewdu R. Wells V. Hebert J. M. Karner C. (2011). A Conserved Pbx-Wnt-P63-Irf6 Regulatory Module Controls Face Morphogenesis by Promoting Epithelial Apoptosis. Dev. Cell. 21 (4), 627–641. 10.1016/j.devcel.2011.08.005 21982646 PMC3199312

[B6] Fisher M. L. Balinth S. Mills A. A. (2020). p63-related Signaling at a Glance. J. Cell. Sci. 133 (17), jcs228015. 10.1242/jcs.228015 32917730 PMC7502597

[B7] Fisher M. L. Kerr C. Adhikary G. Grun D. Xu W. Keillor J. W. (2016). Transglutaminase Interaction with α6/β4-Integrin Stimulates YAP1-dependent ΔNp63α Stabilization and Leads to Enhanced Cancer Stem Cell Survival and Tumor Formation. Cancer Res. 76 (24), 7265–7276. 10.1158/0008-5472.CAN-16-2032 27780825 PMC5161627

[B8] Galoczova M. Coates P. Vojtesek B. (2018). STAT3, Stem Cells, Cancer Stem Cells and P63. Cell. Mol. Biol. Lett. 23, 12. 10.1186/s11658-018-0078-0 29588647 PMC5863838

[B9] Galoczova M. Coates P. Vojtesek B. (2018). STAT3, Stem Cells, Cancer Stem Cells and P63. Cell. Mol. Biol. Lett. 23, 12. 10.1186/s11658-018-0078-0 29588647 PMC5863838

[B10] Gatti V. Bongiorno-Borbone L. Fierro C. Annicchiarico-Petruzzelli M. Melino G. Peschiaroli A. (2019). p63 at the Crossroads between Stemness and Metastasis in Breast Cancer. Ijms 20 (11), 2683. 10.3390/ijms20112683 PMC6600246 31159154

[B11] Gatti V. Fierro C. Compagnone M. La Banca V. Mauriello A. Montanaro M. (2022). ΔNp63-Senataxin Circuit Controls Keratinocyte Differentiation by Promoting the Transcriptional Termination of Epidermal Genes. Proc. Natl. Acad. Sci. U.S.A. 119 (10), e2104718119. 10.1073/pnas.2104718119 35235452 PMC8915885

[B12] Gerbe A. Alame M. Dereure O. Gonzalez S. Durand L. Tempier A. (2019). Systemic, Primary Cutaneous, and Breast Implant-Associated ALK-Negative Anaplastic Large-Cell Lymphomas Present Similar Biologic Features Despite Distinct Clinical Behavior. Virchows Arch. 475 (2), 163–174. 10.1007/s00428-019-02570-4 30953147

[B13] Heichler C. Scheibe K. Schmied A. Geppert C. I. Schmid B. Wirtz S. (2020). STAT3 Activation through IL-6/IL-11 in Cancer-Associated Fibroblasts Promotes Colorectal Tumour Development and Correlates with Poor Prognosis. Gut 69 (7), 1269–1282. 10.1136/gutjnl-2019-319200 31685519

[B14] Jin W. (2020). Role of JAK/STAT3 Signaling in the Regulation of Metastasis, the Transition of Cancer Stem Cells, and Chemoresistance of Cancer by Epithelial-Mesenchymal Transition. Cells 9 (1), 217. 10.3390/cells9010217 PMC7017057 31952344

[B15] Johnson D. E. O'Keefe R. A. Grandis J. R. (2018). Targeting the IL-6/JAK/STAT3 Signalling axis in Cancer. Nat. Rev. Clin. Oncol. 15 (4), 234–248. 10.1038/nrclinonc.2018.8 29405201 PMC5858971

[B16] Koster M. I. Lu S.-L. White L. D. Wang X.-J. Roop D. R. (2006). Reactivation of Developmentally Expressed P63 Isoforms Predisposes to Tumor Development and Progression. Cancer Res. 66 (8), 3981–3986. 10.1158/0008-5472.CAN-06-0027 16618715

[B17] Lin W. Wan X. Sun A. Zhou M. Chen X. Li Y. (2021). RUNX1/EGFR Pathway Contributes to STAT3 Activation and Tumor Growth Caused by Hyperactivated mTORC1. Mol. Ther. - Oncolytics 23, 387–401. 10.1016/j.omto.2021.10.009 34853810 PMC8605091

[B18] Liu B. Yan L. Zhou M. (2019). Target Selection of CAR T Cell Therapy in Accordance with the TME for Solid Tumors. Am. J. Cancer Res. 9 (2), 228–241. 30906625 PMC6405971

[B19] Ma J.-h. Qin L. Li X. (2020). Role of STAT3 Signaling Pathway in Breast Cancer. Cell. Commun. Signal 18 (1), 33. 10.1186/s12964-020-0527-z 32111215 PMC7048131

[B20] Ma J. Meng Y. Kwiatkowski D. J. Chen X. Peng H. Sun Q. (2010). Mammalian Target of Rapamycin Regulates Murine and Human Cell Differentiation through STAT3/p63/Jagged/Notch Cascade. J. Clin. Invest.. 120 (1), 103–114. 10.1172/JCI37964 20038814 PMC2798675

[B21] Mangiulli M. Valletti A. Caratozzolo M. F. Tullo A. Sbisà E. Pesole G. (2009). Identification and Functional Characterization of Two New Transcriptional Variants of the Human P63 Gene. Nucleic Acids Res. 37 (18), 6092–6104. 10.1093/nar/gkp674 19700772 PMC2764424

[B22] Murray-Zmijewski F. Lane D. P. Bourdon J.-C. (2006). p53/p63/p73 Isoforms: an Orchestra of Isoforms to Harmonise Cell Differentiation and Response to Stress. Cell. Death Differ. 13 (6), 962–972. 10.1038/sj.cdd.4401914 16601753

[B23] Peng W. Chang M. Wu Y. Zhu W. Tong L. Zhang G. (2021). Lyophilized Powder of Mesenchymal Stem Cell Supernatant Attenuates Acute Lung Injury through the IL-6-p-STAT3-p63-JAG2 Pathway. Stem Cell. Res. Ther. 12 (1), 216. 10.1186/s13287-021-02276-y 33781349 PMC8008635

[B24] Rufini S. Lena A. M. Cadot B. Mele S. Amelio I. Terrinoni A. (2011). The Sterile Alpha-Motif (SAM) Domain of P63 Binds *In Vitro* Monoasialoganglioside (GM1) Micelles. Biochem. Pharmacol. 82 (10), 1262–1268. 10.1016/j.bcp.2011.07.087 21820419

[B25] Sayan B. S. Sayan A. E. Yang A. L. Aqeilan R. I. Candi E. Cohen G. M. (2007). Cleavage of the Transactivation-Inhibitory Domain of P63 by Caspases Enhances Apoptosis. Proc. Natl. Acad. Sci. U.S.A. 104 (26), 10871–10876. 10.1073/pnas.0700761104 17581882 PMC1904122

[B26] Sethi I. Sinha S. Buck M. J. (2014). Role of Chromatin and Transcriptional Co-regulators in Mediating P63-Genome Interactions in Keratinocytes. BMC Genomics 15 (1), 1042. 10.1186/1471-2164-15-1042 25433490 PMC4302094

[B27] Siersbæk R. Scabia V. Nagarajan S. Chernukhin I. Papachristou E. K. Broome R. (2020). IL6/STAT3 Signaling Hijacks Estrogen Receptor α Enhancers to Drive Breast Cancer Metastasis. Cancer Cell. 38 (3), 412–423. 10.1016/j.ccell.2020.06.007 32679107 PMC7116707

[B28] Soares E. Zhou H. (2018). Master Regulatory Role of P63 in Epidermal Development and Disease. Cell.. Mol. Life Sci. 75 (7), 1179–1190. 10.1007/s00018-017-2701-z 29103147 PMC5843667

[B29] Sun H.-J. Xiong S.-P. Cao X. Cao L. Zhu M.-Y. Wu Z.-Y. (2021). Polysulfide-mediated Sulfhydration of SIRT1 Prevents Diabetic Nephropathy by Suppressing Phosphorylation and Acetylation of P65 NF-Κb and STAT3. Redox Biol. 38, 101813. 10.1016/j.redox.2020.101813 33279869 PMC7718489

[B30] Tošić I. Frank D. A. (2021). STAT3 as a Mediator of Oncogenic Cellular Metabolism: Pathogenic and Therapeutic Implications. Neoplasia 23 (12), 1167–1178. 10.1016/j.neo.2021.10.003 34731785 PMC8569436

[B31] Wei S. X. (2020). Research Progress on Mechanism and Clinical Evidence of Peroxisome Proliferator Activated Receptor Gamma Agonist in the Treatment of Lung Cancer[J]. Pract. J. Cardiac Cereb. Pneumal Vasc. Dis. 28(8):136–140. 10.3969/j.issn.1008-5971.2020.08.027

[B32] Wu J. Bergholz J. Lu J. Sonenshein G. E. Xiao Z.-X. J. (2009). TAp63 Is a Transcriptional Target of NF-Îºb. J. Cell.. Biochem. 109 (4), a–n. 10.1002/jcb.22449 PMC2873845 20052674

[B33] Yang A. Kaghad M. Wang Y. Gillett E. Fleming M. D. Dötsch V. (1998). p63, a P53 Homolog at 3q27-29, Encodes Multiple Products with Transactivating, Death-Inducing, and Dominant-Negative Activities. Mol. Cell. 2 (3), 305–316. 10.1016/s1097-2765(00)80275-0 9774969

[B34] Yang L. Shi P. Zhao G. Xu J. Peng W. Zhang J. (2020). Targeting Cancer Stem Cell Pathways for Cancer Therapy. Sig Transduct. Target Ther. 5 (1), 8. 10.1038/s41392-020-0110-5 PMC7005297 32296030

[B35] Zou S. Tong Q. Liu B. Huang W. Tian Y. Fu X. (2020). Targeting STAT3 in Cancer Immunotherapy. Mol. Cancer 19 (1), 145. 10.1186/s12943-020-01258-7 32972405 PMC7513516

